# Aircraft sound exposure leads to song frequency decline and elevated aggression in wild chiffchaffs

**DOI:** 10.1111/1365-2656.13059

**Published:** 2019-08-21

**Authors:** Andrew D. Wolfenden, Hans Slabbekoorn, Karolina Kluk, Selvino R. de Kort

**Affiliations:** ^1^ Ecology and Environment Research Centre, Department of Natural Sciences Manchester Metropolitan University Manchester UK; ^2^ School of Life Science University of Nottingham Nottingham UK; ^3^ Institute of Biology Leiden (IBL) Leiden University Leiden the Netherlands; ^4^ Manchester Centre for Audiology and Deafness (ManCAD) Manchester University Manchester UK

**Keywords:** aggression, Aircraft noise, bird song, masking, *Phylloscopus collybita*

## Abstract

1. The ubiquitous anthropogenic low‐frequency noise impedes communication by masking animal signals. To overcome this communication barrier, animals may increase the frequency, amplitude and delivery rate of their acoustic signals, making them more easily heard. However, a direct impact of intermittent, high‐level aircraft noise on birds’ behaviour living close to a runway has not been studied in detail.
2. We recorded common chiffchaffs *Phylloscopus collybita* songs near two airports and nearby control areas, and we measured sound levels in their territories at Manchester Airport. The song recordings were made in between aircraft movements, when ambient sound levels were similar between airport and control populations. We also conducted playback experiments at the airport and a control population to test the salience of airport, and control population specific songs.
3. In contrast to the general pattern of increased song frequency in noisy areas, we show that common chiffchaffs at airports show a negative relationship between noise exposure level and song frequency.
4. Experimental data show that chiffchaffs living near airports also respond more aggressively to song playback.
5. Since the decrease in song frequency results in increased overlap with aircraft noise, these findings cannot be explained as an adaptation to improve communication. The increased levels of aggression suggest that chiffchaffs, like humans, might be affected behaviourally by extreme noise pollution. These findings should influence environmental impact assessments for airport expansions globally.

## Graphical abstract

This paper shows that birds sing at a lower frequency and are more aggressive, when exposed to aircraft noise. The lower song frequency cannot be explained as an adaptation to the local sound environment. Instead, the data are consistent with the birds suffering from noise‐induced hearing loss.

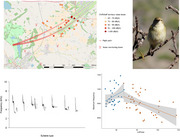

## 1 INTRODUCTION

Animals, like humans, are negatively affected by the global increase in anthropogenic noise levels (Barber, Crooks, & Fristrup, 2010; Shannon et al., 2016). For wildlife, anthropogenic noise leads to displacement, disrupts parent–offspring communication, increases stress‐related hormone levels and vigilance behaviour and changes communication systems (Barber et al., 2010; Kight & Swaddle, 2011; Rich & Romero, 2005). Noise exposure in humans has been linked to hearing loss, tinnitus, hypertension, sleep deprivation and increased stress levels (Basner et al., 2014; Huss, Spoerri, Egger, & Roosli, 2010; Stansfeld & Shipley, 2015). Anthropogenic noise at the current level and scale is a pervasive (Buxton et al., 2017) and relatively novel selection pressure that is projected to increase with human population expansion (Barber et al., 2010). The capacity of animal species to adapt to this novel selection pressure affects their distribution, which contributes to their success as urban adapters (Slabbekoorn, 2013).

Anthropogenic noise sources differ in their acoustic structure and temporal presence, and they may have different effects on wildlife (Gill, Job, Muyers, Naghshineh, & Vonhof, 2015). Motorways produce diel patterns of peaks and troughs in sound level and reach maximum levels of approximately 65 dB(A) along the linear structure of the road with the spectral energy concentrated below 2 kHz (Halfwerk, Holleman, Lessells, & Slabbekoorn, 2011b). Industries, such as resource extraction and construction, usually provide a point source of noise and thus affect restricted areas. However, their sound levels can reach up to 75–90 dB(A) (Habib, Bayne, & Boutin, 2007). Trains and aircrafts produce intermittent noise, interspersed with periods of relative quiet. Aircraft movements can reach extreme noise levels of over 100 dB(A), at 100 m distance from an aircraft taking off (Goudie & Jones, 2004).

Noise interferes with acoustic communication between animals through the masking of their signals. Masking decreases the signal‐to‐noise ratio (SNR) of an acoustic signal, reducing the available transmission distance and thus making communication less effective (Lohr, Wright, & Dooling, 2003). Animals have essentially three strategies to counteract the masking effect of anthropogenic noise. They can increase the amplitude of their signals and thus increase the SNR, a process known as the Lombard effect and observed in many animals (Brumm, 2004). The capacity to increase the SNR depends on the level of masking noise and the flexibility of animal species to increase their signal amplitude. A second strategy involves changing the delivery time of the signals to avoid the temporal overlap between signal and noise (Arroyo‐Solís, Castillo, Figueroa, López‐Sánchez, & Slabbekoorn, 2013; Fuller, Warren, & Gaston, 2007). A third strategy relies on the species’ capacity to change the acoustic structure of their signals to facilitate masking release. When exposed to low‐frequency anthropogenic noise, animals may increase the frequency of their acoustic signals, rather than the amplitude as in the Lombard effect (Slabbekoorn & Peet, 2003), presumably to reduce the effect of masking. Noise‐dependent upward frequency shifts have been observed for a wide range of bird species (Slabbekoorn, 2013).

To date, only one study has addressed the impact of aircraft noise on bird song structure. In contrast to other anthropogenic noise sources, the frequency of blackbird (*Turdus merula*) songs did not differ between quiet and airport populations (Sierro, Schloesing, Pavon, & Gil, 2017). An explanation for this is that frequency adjustment is not an effective strategy as aircraft sound levels may exceed the capacity for most if not all animals to increase the signal‐to‐noise ratio sufficiently to be heard. Birds move away from continuous noise at airfields (Swaddle, Moseley, Hinders, & Smith, 2016) and reduce singing when their songs are masked by aircraft noise above 78 dB(A) SPL (Dominoni, Greif, Nemeth, & Brumm, 2016). However, aircraft noise is intermittent, and thus, birds can use quiet phases in between aircraft movements to communicate. This strategy would require no spectral adjustment to the song to maintain signal efficacy. Indeed, when exposed to aircraft noise, birds adjust the onset of dawn singing to avoid peak aircraft‐movement times and reduce overlap with aircraft noise (Dominoni et al., 2016; Gil, Honarmand, Pascual, Perez‐Mena, & Macias Garcia, 2014; Sierro et al., 2017). Nevertheless, many birds remain in their territories throughout the daily cycle of aircraft movements and continue singing even in peak aircraft‐movement times.

Here, we compared song structure of common chiffchaffs (*Phylloscopus collybita*) at two different airports (Manchester and Amsterdam) and two control sites. We also measured aircraft sound levels in chiffchaff territories at Manchester Airport and related these to song characteristics of individual chiffchaffs. The song recordings at the airports were made in between aircraft movements. Having demonstrated that the songs of chiffchaffs exposed to aircraft noise decreased in spectral parameters, we then proceeded to investigate whether the spectral change was biologically relevant. Spectral and temporal properties of bird song convey information about body condition, status and motivation to fight (Gil & Gahr, 2002) and play an important role in mate attraction and territory defence (Catchpole & Slater, 2003). When these parameters change, this may also affect the signal value of the songs (Halfwerk, Bot, et al., 2011a; de Kort, Eldermire, Cramer, & Vehrencamp, 2009a) in the context of sexual selection. This raised the question whether the airport songs that differ in acoustic properties from the control songs are effective in territory defence. To that end, we conducted playback experiments at Manchester Airport and a nearby control location.

## 2 MATERIALS AND METHODS

### 2.1 Study site and species

The main study sites were Manchester Airport, UK (53.351039, −2.279860) and Woolston Eyes nature reserve (53.389471, −2.528626) as a control site, 20 km to the southeast. Additional sound recordings were obtained from Schiphol Airport, Netherlands (52.317438, 4.823373), and Meijendel nature reserve (52.126934, 4.340512) as a control site, approximately 50 km to the southwest from the airport. Manchester Airport has approximately 490 aircraft movements on two runways a day (CAA 2015), while Schiphol Airport has approximately 1,200 aircraft movements on six runways a day (Airport Council International, 2016). At Manchester Airport, this study focused on the area around runway 2 that contributed 85% of all aircraft movements in 2014 (MAG Departure information pack 2017). All study sites are characterized by scrublands surrounding small patches of broadleaf woodland, with willow (*Salix *sp.), hazel (Corylus), sycamore *(Acer pseudoplatinus*) and oak (*Quercus *sp.) being the dominant tree species.

Chiffchaffs are summer migrants to Europe, with the first males usually arriving in March. Males defend their territories by singing from strategic positions throughout the breeding season, which typically concludes at the end of June. Male chiffchaffs mediate social interactions by modifying temporal and spectral song parameters. Fighting ability is signalled with a relatively low peak frequency (Linhart, Slabbekoorn, & Fuchs, 2012), while duration of songs signals motivation to fight (Linhart, Jaska, Petruskova, Petrusek, & Fuchs, 2013). Chiffchaffs can shift song frequencies immediately in response to anthropogenic noise (Verzijden, Ripmeester, Ohms, Snelderwaard, & Slabbekoorn, 2010). The start of the dawn chorus in chiffchaffs does not differ between quiet sites and those exposed to aircraft noise (unpublished data).

### 2.2 Noise measures

Noise level measurements for UK sites were obtained between 06:30 and 12:00 from March to June 2014**.** To obtain sound levels, the maximum level with A‐weighted frequency response and fast time constant (LAFmax) for each of the chiffchaff territories in the UK was measured using a sound level meter (Precision Gold N05CC), set at 1.5 m from the ground. In addition, average ambient noise levels (LAeq (*t*)) were obtained by recording sound levels every second for a 10‐min period using a class 2 industry standard sound level meter (Casella CEL‐246, Fast response, A weighted). Where possible, the noise level meters were tripod mounted at a height of 1.5 m, facing vertically upwards directly underneath the singing post. Where a tripod could not be positioned directly underneath the singing post, the closest open space was selected. Noise levels were compared between sites with a two‐tailed, independent *t* test. Additionally, LAeq(t) were compared between airport and control sites in between aircraft movements. The 10‐min sound level recordings consist of 600 measurements, and each measurement that exceeded background noise levels and could be attributed to aircraft movement (generally above 60 dB(A)) was removed to generate LAeq for airport sites without aircraft noise. Sound level data for the sites in the Netherlands were not collected for this aspect of the study.

### 2.3 Song recording and analysis

Song recordings were made between 06:30 and 12:00 from March 17 to June 30 2014 near Manchester Airport and Woolston Eyes nature reserve on alternate days. The distance between the territories of recorded individuals and the runway ranged between 180 m and 2,100 m at the Manchester airport site. The recordings for Schiphol Airport and Meijendel nature reserve were made in May 2015. Each 10‐min recording session was preceded by a 5‐min habituation period to reduce the effect of observer presence on singing behaviour. To reduce the chance of recording the same individual twice, no song recordings were made within 200 m of another recorded individual. This distance between territories is twice the recorded territory size in chiffchaffs (Rodrigues, 1998). In some cases, while recording one individual, another bird was observed singing and in that case the second bird may have been recorded closer than 200 m. Each individual chiffchaff was recorded from a maximum distance of 10 m, and the bird was always in sight of the recorder. Recordings were made using a Sennheiser ME67 microphone and a Marantz PMD661 MKII digital recorder (sampling frequency: 44,100 Hz; 16 bit; WAV format). For each recorded individual, a random sample of ten songs was selected from the 10‐min recording using the sample function, without replacement, in R (R Core Team, 2016). Because of the inherent difficulties in obtaining accurate frequency measurements during noise events (Brumm, Zollinger, Niemelä, Sprau, & Schielzeth, 2017; Verzijden et al., 2010), only songs recorded in between aircraft movements were used for song analysis. This is important to note because this means that during the song recording, ambient sound levels were not affected by aircraft movements. Four spectral and three temporal parameters for each song were measured using the automatic parameter measurement feature in Avisoft‐SASLab Pro version 4.3 (Avisoft bioacoustics, Berlin, Germany). The automatic parameter measurement feature allows for objective measurements across the different recordings and is independent of recording quality. For element separation, an automatic single threshold of −21 dB was used with a hold time of 100 ms (spectrogram settings: Hamming window FFT size = 512, overlap 50%). The spectral parameters measured were maximum, minimum, peak frequency and frequency bandwidth, and the temporal parameters were syllable and song duration, and number of syllables.

Two types of analyses were conducted on the song parameters. The first tests for song structural differences between airport and control birds. Song parameters were compared as a function of site (airport or control site) using linear mixed effects models with country (UK or Netherlands) as a random factor. Mixed effects models were compared to null models with no random effects using an ANOVA. Models were validated by inspection of residual plots. Post hoc Tukey tests were used to further explain any significant results. All *p* values reported are adjusted values following sequential Bonferroni correction for multiple testing (Rice 1989).

The second type of analysis tests for a relationship between LAFmax as measured in an individual's territory and the 6 song parameters. This analysis was done separately for the Manchester airport population and the control population, and did not include the data from the Netherlands. For the comparison within sites, MANOVA models with LAFmax and Julian date as independent factors were used. Julian date was included to control for seasonal variation in song parameters (Vehrencamp, Yantachka, Hall, & Kort, 2013). Model selection was based on the lowest Akaike information criterion (AIC) value (Zuur, Ieno, Walker, Saveliev, & Smith, 2009).

### 2.4 Syllable type

**Figure 1:**
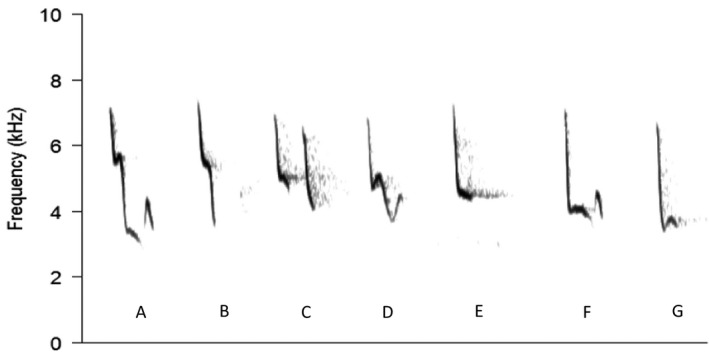
Spectrogram of all syllable types of chiffchaff songs (*Phylloscopus collybita*) recorded in the UK. Syllables are order ranked from highest to lowest peak frequency. Following discriminant function analysis, syllable types C and D were merged. Figure spectrogram settings: Hamming window, FFT size 256 and overlap of 87.5%

Initial visual inspection of spectrograms of chiffchaff songs for both UK populations suggested 7 different syllable types (Figure 1). Discriminant function analysis (DFA) was used for objective categorization of syllable types. Twenty random samples of each syllable type were selected using the R sample function with no replacement. A discriminant function separated the syllable types based on maximum, minimum, and peak frequency and syllable length for a subset (*n* = 10) of each syllable type. The function was then used to categorize the remaining 10 syllables for each type. The proportion of observed syllable types was compared to the proportion of predicted syllable types to test the accuracy of the DFA. Two syllable types (c and d, see Figure 1) were not discriminated by the function, and these were merged, leaving 6 distinct syllable types. The categorization was then used to assess the average proportion of each syllable type within the songs of chiffchaffs at the airport and control sites. The difference between the two sites was tested with a Wilcoxon signed‐ranks test.

### 2.5 Playback procedure

Playback trials were conducted between 6:00 and 11:00 from March 31 to April 19 2015 in the UK sites. A remotely controlled loudspeaker (Fox Pro Fury, www.gofoxpro.com) was placed in a tree at approximately 1.8 m height within the territory of a subject. All observations were conducted from a camouflaged pop‐up hide positioned approximately 10 m from the loudspeaker, which the observer entered at least five minutes before start of playback.

### 2.6 Playback design

The stimuli were created from songs recorded from 22 males (*N* = 11 Manchester Airport, *N* = 11 control site). Songs were randomly selected using the sample function with replacement in R (R Core Team, 2016) from a database of recordings made in 2014. The songs of the birds at the airport contain fewer high notes (i.e. note “A”, see Figure 1 and results) and more low notes (note “G”) than the songs of control birds. Thus, to create pairs of stimuli that were identical, except for the proportion of high and low notes we replaced the high A‐type syllable with the low G‐type syllable from the same song to create an airport‐type song. Similarly, to create a control‐type stimulus, we replaced the low G‐type syllable with the high A‐type syllable from the same song. Only songs that contained both airport‐type (G‐type) and control‐type syllables (A‐type) were used for stimulus preparation, and both control and airport population contributed the same number (11) of original recordings.

Song files were band‐pass filtered (1,000–9,000 Hz), and the amplitude was normalized to 90% of the maximum amplitude in Avisoft‐SASLab (Specht. R, Berlin, Germany). The procedure ensured that song lengths and syllable rates of the manipulated songs did not differ from those for the original recordings or between airport‐type or control‐type stimuli within a pair of stimuli. Each subject was exposed to a pair of stimuli derived from the same original song, and the stimuli only differed in the proportion of high/low notes. This procedure precludes other song variables, such as duration or delivery rate, to affect the response of the birds.

Each playback trial was divided into three 120‐s observation periods. The pre‐playback period (120 s of silence) was the baseline period for that subject, followed by two exposure periods (120 s) consisting of 30 s of playback followed by 90s of observation each. The order of playback stimulus type (airport and control) alternated between subjects. Behavioural responses were recorded using a data logging application (SpectatorGo! http://www.biobserve.com/products/spectator_go/) on a touch screen device (IPod touch: www.apple.com). Subject responses were assessed with three behavioural variables: (a) attack = the number of times the individual came into physical contact with the loudspeaker, (b) flight = the number of times the subject flew within 2 m of the loudspeaker and (c) song = the number of times the subject vocalized. Each playback trial was conducted on a different subject. Birds in adjacent territories were not tested in the same 24‐hr period to avoid carryover effects. All subjects at the airport (*N* = 33) and control site (*N* = 33) were tested for both stimuli.

### 2.7 Statistical analyses

Generalized linear models assuming Poisson distributions and using a loglink function were built to assess the effect of stimulus type on the response measures. To control for effect of seasonality or location of original recording, Julian date and recording site (airport or control) were included as additional independent variables. Model selection was based on Akaike's information criteria (AIC) for each model (Zuur et al. 2009). Sequential Bonferroni corrections were applied to control for the increased probability of type 1 errors as a result of multiple testing (Rice, 1989). Crossover (Diaz‐Uriarte, 2002) and order effects were tested for with Mann–Whitey U tests and Wilcoxon signed‐rank tests, respectively.

## 3 RESULTS

### 3.1 Sound exposure levels

**Figure 2:**
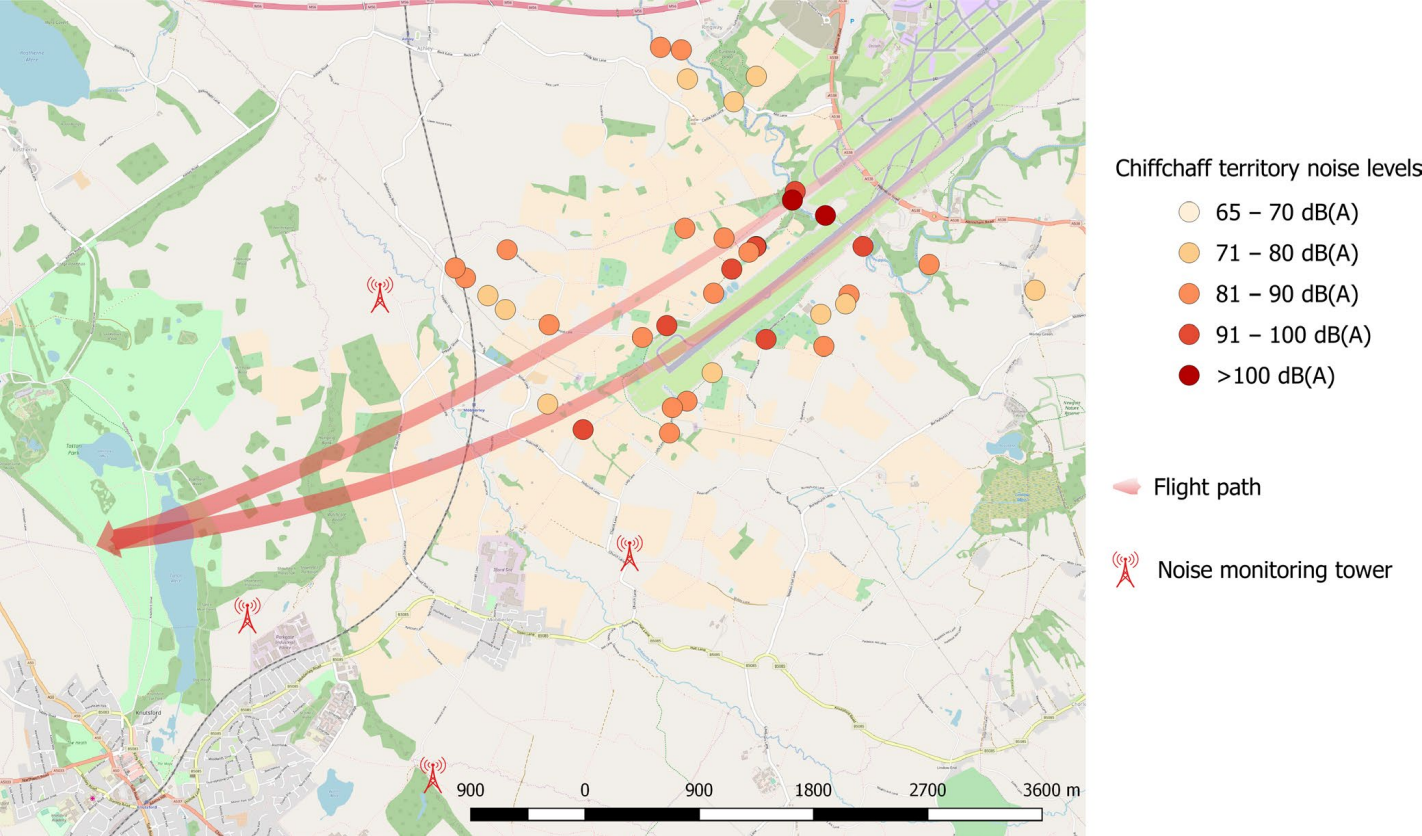
Map of the study area around Manchester airport indicating the location and sound level of the chiffchaff territories sampled for this study. The main aircraft flight path from each runway and the location of noise monitoring towers are indicated

Sound levels generated by aircraft movements measured at chiffchaff territories at Manchester Airport varied between LAFmax 67 and 118 dB(A) (mean LAFmax = 81.93 dB(A) ± SDE = 9.11, see Figure 2), while at the control site sound levels varied between LAFmax = 42 and 67.3 dB(A) (mean LAFmax = 57.13 dB(A) ± SDE 4.57). The mean LAFmax sound levels differed between the airport and control territories (*T* = 12.70, *p *= <0.001). The LAeq sound levels at the airport territories measured over a 10‐min period that included aircraft noise varied between 51 and 67 dB(A) (mean LAeq = 58.5 dB(A) ± SDE = 4.51). The LAeq recorded at Manchester airport territories in between aircraft movements ranged from 43.0 to 56.5 dB(A) (mean LAeq = 47.91 dB(A) ± 3.45 SDE) and did not differ to ambient noise levels at the control site (control min LAeq = 42.90, control max LAeq = 51.0, control mean LAeq = 46.42 ± 2.59 SDE; *t* test: T = 1.72, *p* = 0.09).

### 3.2 Song structure at airports and control site

**Figure 3:**
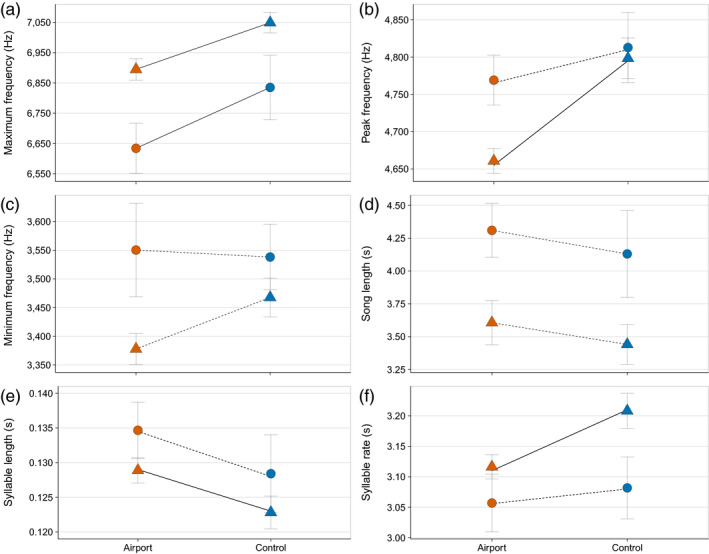
Comparison of mean (±*SEM*) of six chiffchaff (*Phylloscopus collybita*) song parameters recorded at two airports (orange, Manchester *N* = 38, Schiphol, *N* = 18) and quiet control sites (blue, Woolston eyes nature reserve *N* = 30, and Meijendel nature reserve *N* = 18) in two countries (triangles = UK, circles = the Netherlands). Unbroken lines indicate significant differences between airport and control sites within a country, while dotted lines indicate non‐significant differences

When comparing the average song parameters between airport and control populations, we included individuals from Manchester Airport (*N* = 38) and control site (*N* = 30) and additional recordings from Schiphol Airport (*N* = 18) and control site (*N* = 15). Chiffchaffs at airport sites show a lower average song Maximum frequency (*F*_3,100_ = 9.86, *p *= <0.001). This was replicated at the population level, at the two widely separated airports (UK Airport vs. UK control: *Z* = 2.461, *p* = 0.042; NL airport vs. NL control: *Z* = 2.741, *p* = 0.024) (Figure 3). There was also significant variation in peak frequency (*F*_3,100_ = 8.77, *p *= <0.001) between sites; airport birds in the UK used lower peak frequencies than control birds (UK control vs. UK Airport: *Z* = 2.461, *p* = <0.001). In the Netherlands, no difference in peak frequency between the control and the airport population was detected (NL control vs. NL airport: *Z* = 2.741, *p* = 0.355), although the numerical difference was in the same direction (Figure 3). Overall, there was significant variation in minimum frequency between sites (*F*_3,100_ = 8.77, *p* = 0.03). Post hoc analyses showed that this difference could be attributed to NL airport and UK control sites (*Z* = −2.802, *p* = 0.031). Overall Chiffchaffs in the Netherlands used lower maximum frequencies but higher peak frequencies than those in the UK (Figure 3), a level of geographic variation that is not unusual between distant populations of the same species (Podos & Warren, 2007; Slabbekoorn & Den Boer‐Visser, 2006). Syllable rate differed between sites (*F*_3,100_ = 24.18, *p* = 0.001), while this variation can be accounted for by differences between sites in different countries, significant differences in syllable length were detected between UK control and UK airport sites (Z = 2.351, *p* = 0.038). There was no significant variation in song length between any of the sites (*F*_3,100_ = 4.024, *p* = 0.09). The variables “Number of syllables” and “Frequency bandwidth” were removed from further analysis because they were highly correlated with “Song length” and “Syllable duration” (*r* > 0.80) and with “Peak frequency” and “Maximum frequency” (*r* > 0.60), respectively.

### 3.3 Song structure and aircraft noise levels

**Figure 4:**
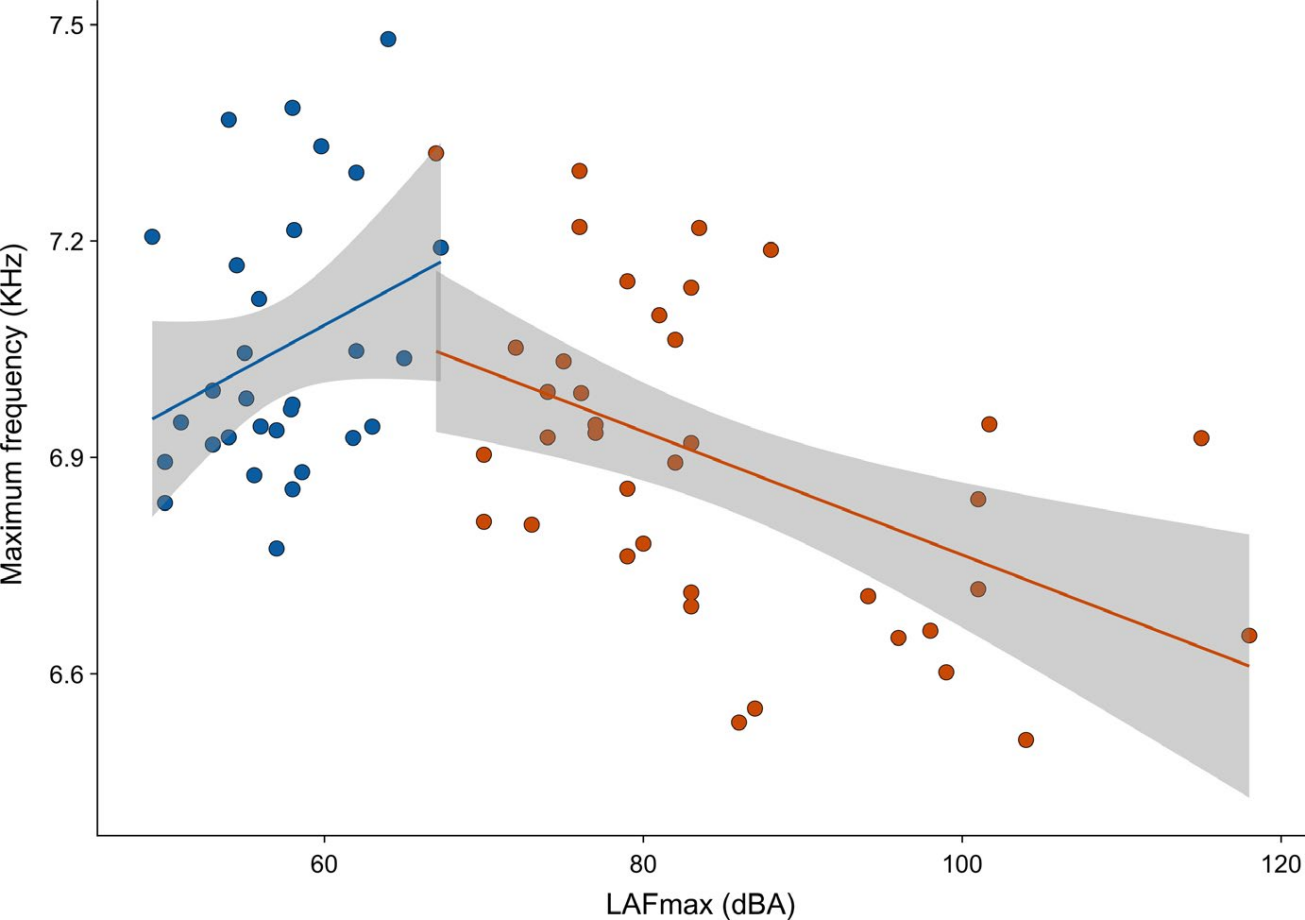
Maximum frequencies of the songs of individual common chiffchaffs (*Phylloscopus collybita*) around Manchester Airport (orange) and control site (blue). Maximum frequencies varied over a range of about 1,000 Hz and were correlated with the maximum sound level (LAFmax) measured at the territory. Blue dots represent birds from the control site and show a positive correlation between the maximum song frequency and the maximum noise level (LAFmax) at their territory. Red dots represent airport birds and show a negative correlation between the maximum song frequency and the maximum noise level at their territory

A detailed spectral analysis of songs at the individual level around Manchester Airport (*N* = 38) showed a significant decrease in the maximum frequency of chiffchaff songs with an increase in LAFmax (*N* = 38, *F*_1,37_ = 12.907, *p* = 0.001). There was no effect on LAFmax on any other song parameters in the airport population (Table 1). Congruent with other studies, there was a positive correlation with minimum frequency and LAFmax detected at the quiet control site (*N* = 30, *F*_1,29_ = 12.907, *p* = 0.001; Figure 4). Control birds also sang at a slower rate as LAFmax increased (*N* = 30, *F*_1,29_ = 8.808, *p* = 0.006). There was no effect of LAFmax on any other song parameter in the control population. Julian date had no effect on any of the temporal or spectral variables at either airport or control sites (Table 1). The reduced maximum frequency in the songs of the airport population results from a lower percentage of high‐frequency syllable type A (airport = 12.7%, control = 20.4%, *W* = 418.5, *p* = 0.046) and higher percentage of low‐frequency syllable type G (airport = 18%, control = 8%, *W* = 761, *p* = 0.014) in the songs, rather than a complete downward spectral shift of the songs. In other respects, the syllable repertoires and relative syllable use were identical between the two populations.

**Table 1:** Test statistics for MANOVA models used to explore the effects of LAFmax and season (Julian date) on common chiffchaff (*Phylloscopus collybita*) song parameters at Manchester Airport (*N* = 38) and Woolston Eyes nature reserve (control, *N* = 30)

Parameters	Effect	Airport		Control	
		*F*	*p*	*F*	*p*
MaxF	LAFmax	**12.907**	**0.0001**	2.647	0.115
	Julian Date	6.078	0.018	0.021	0.885
MinF	LAFmax	0.152	0.699	**4.554**	**0.042**
	Julian Date	0.500	0.482	0.159	0.693
PeakF	LAFmax	0.373	0.545	0.850	0.364
	Julian Date	0.338	0.565	0.131	0.720
Syll. Length	LAFmax	0.109	0.743	**8.808**	**0.006**
	Julian Date	3.535	0.068	0.395	0.535
Syll. Rate	LAFmax	0.498	0.498	8.542	0.007
	Julian Date	3.365	0.075	2.487	0.126
Song length	LAFmax	0.001	0.980	0.004	0.945
	Julian Date	0.020	0.890	1.232	0.277

Abbreviations: MaxF, Maximum Frequency (kHz); MinF, Minimum Frequency (kHz); PeakF, Peak Frequency (kHz); Syll. Length, duration of syllable (s); Syll. Rate, Number of syllables/ (s), Song length = Duration of song (s).

Bold values indicate significance following *p*‐value adjustment for multiple testing.

### 3.4 Playback results

**Figure 5:**
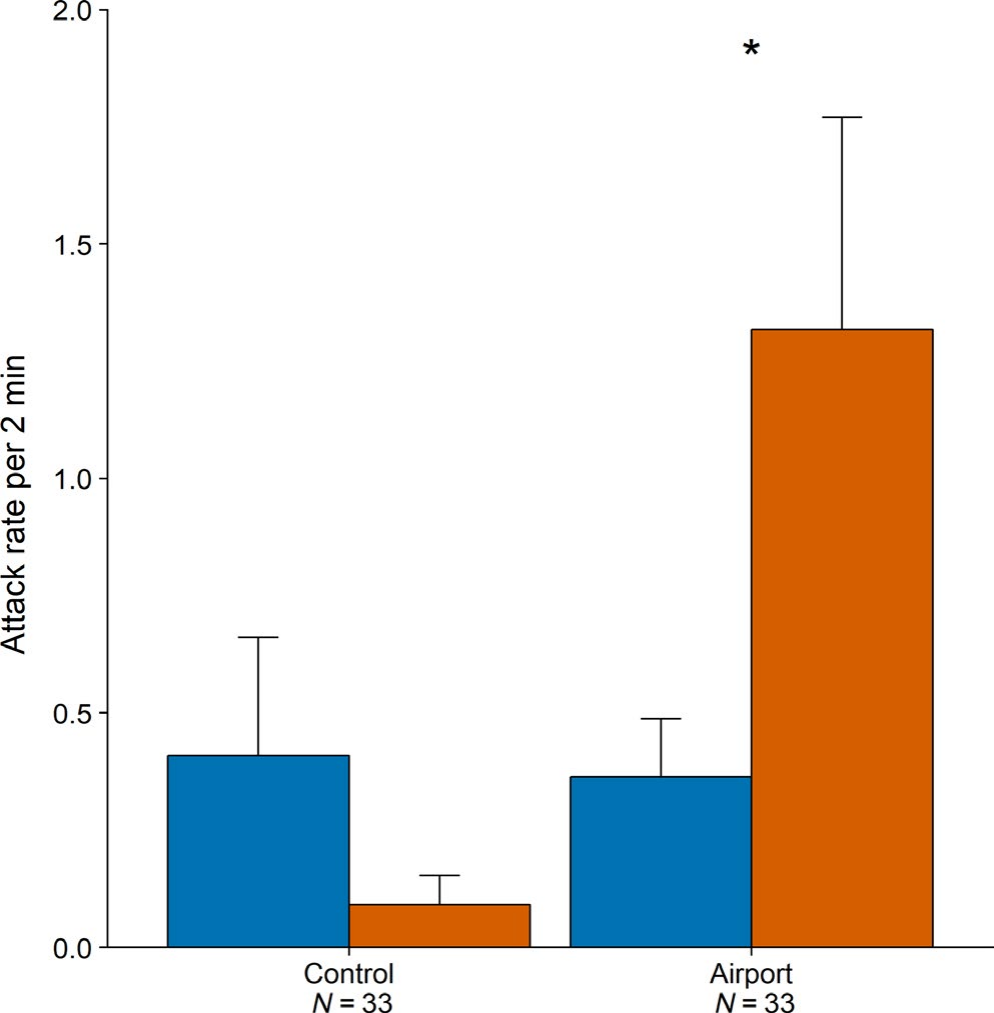
Average number of attacks per 120 s by common chiffchaffs on the playback loudspeaker broadcasting either control (blue) or airport (orange) type stimuli for the control population (left) and the airport population (right). The '*' indicates significant difference in number of attacks on loud speaker between following playback of airport and control stimuli

Both airport and control stimuli elicited a strong response based on two behavioural response measures in both the airport and control population. The airport population responded with a reduced number of songs (control stimulus: *n* = 33, *Z* = −4.64, *p* < 0.001; airport stimulus: *n* = 33, *Z* = −4.83, *p* < 0.001) and an increase in approach to the playback loudspeaker (control, *n* = 33, *Z* = 4.97, *p* < 0.001; airport: *n* = 33, *Z* = 3.25, *p* = 0.001), compared to baseline behaviour during the pre‐playback period. The control population responded similarly with a smaller number of songs (control: *n* = 33, *Z *= −3.097, *p* = 0.002; airport: *n* = 33, *Z *= −1.072, *p* = 0.006) and an increase in approach to the playback loudspeaker (control: *n* = 33, *Z* = 3.57, *p* < 0.001, airport: *n* = 33, *Z* = 2.09, *p* = 0.001). However, although both populations clearly responded to the stimuli, they did not show a difference in response to the two playback stimuli for these 2 response parameters (all *p* > 0.05). Nevertheless, at the airport a fivefold higher number of individuals (25/33) physically attacked the loudspeaker following playback compared to control birds (5/33, Fisher's exact test: airport *N* = 33, control, *N* = 33, *p* < 0.001; Figure 5). The airport population attacked the playback speaker more in response to airport than control stimuli (*Z* = 2.49, *p* = 0.03), a pattern not displayed by the control population. The difference in response is not an immediate result of exposure to aircraft noise, as all trials were conducted when there were no aircraft movements.

## 4 DISCUSSION

Chiffchaffs holding territories near Manchester airport runway 2 are exposed to extreme sound levels, frequently exceeding LAFmax of 110 dB(A). These chiffchaffs sing songs containing more low‐frequency syllables with lower maximum and peak frequency and a slower song rate than nearby control populations, and the spectral changes are replicated for chiffchaffs living near Schiphol Airport in the Netherlands. In addition, the maximum song frequency decreases with increasing noise levels as measured in the birds’ territories, while the control population shows a positive relationship with more moderate territorial noise levels, congruent with other studies (Slabbekoorn, 2013). Both airport and control populations respond strongly and indiscriminately to both airport‐type (low frequency) and control‐type (high frequency) songs in two response parameters, showing that both stimulus types are equally salient to the birds. However, a third response measure shows that the airport population is more aggressive, with 5 times more individuals physically attacking the playback loudspeaker than the control population.

The sound levels measured at Manchester Airport in this study are similar to those in a study on the impact of aircraft noise on behaviour of harlequin ducks (*Histrionicus histrionicus*) (Goudie & Jones, 2004). Our measurements appear higher than those reported by the Manchester Airport Noise Information System (MANTIS, see Table 1). However, the MANTIS system reports Lden rather than LAFmax. In addition, although Manchester Airport has 13 noise level meters around the airport, the closest of these (Mobberley primary school 53.319662, −2.31352) is approximately 1.4 km away from the nearest runway edge, while our closest measurement is 186 m from the runway. The U.S. department of Transportation, Federal Aviation Administration provides modelled noise level data per aircraft type (Burleson, 2002). The loudest aircraft, the Boeing B‐747–100, is estimated to produce 100.5 dB(A), while the quietest, a Cessna 152, is estimated to produce 55 dB(A) at a distance of 6.5km from the start of the take‐off roll. Given that halving the distance from the aircraft equals a 6 dB increase in sound level, these values correspond to 130 dB(A) and 85.2 dB(A) at 200m, respectively, which is in line with our measurements.

The lower song frequency and delivery rate of chiffchaffs at airports contrast to that reported in most other studies on the impact of anthropogenic noise on birdsong structure. Often‐replicated results show that birds typically increase the spectral frequency and delivery rate of their acoustic signals under a regime of more moderate anthropogenic noise (Patricelli & Blickley, 2006; Slabbekoorn, 2013; Slabbekoorn & Den Boer‐Visser, 2006). One explanation for the increase in song frequency parameters in birds exposed to noise is that it releases the acoustic signals from masking by low‐frequency anthropogenic noise. However, the decrease in song maximum and peak frequency does not lead to masking release during aircraft movements. If anything, by reducing the frequency of their songs closer to the frequency of maximum power in the aircraft sound, they are increasing the masking effect. Additionally, the decrease in spectral parameters in the songs of the chiffchaffs at airports is not a direct response to noise exposure (Verzijden et al., 2010). Recordings were made in between aircraft movements, when ambient sound levels were comparable to those at control sites. It is possible that chiffchaffs do increase the frequency of their songs during aircraft movements, but this was impossible to measure as the aircraft noise precluded spectral measurements in our recordings (Brumm et al., 2017; Verzijden et al., 2010).

If the birds would respond to aircraft noise by singing louder (which we did not measure) as predicted by the Lombard effect, we would expect an increase rather than a decrease in frequency values (Nemeth et al., 2013). In addition, the level of aircraft noise close to the airport runway is so high that the excitation patterns in the bird's cochlea, which is governed by similar mechanisms as mammals (Saunders, Rintelmann, & Bock, 1979), will not produce peaks that would allow detection of the signal in aircraft noise (Wong, Miller, Calhoun, Sachs, & Young, 1998; Zilany, Bruce, Nelson, & Carney, 2009). Instead, the excitation pattern would become almost flat across a wide frequency range, thus making it impossible to detect any additional signal that might be present at the same time as the aircraft noise; that is, aircraft noise will most likely lead to complete masking of the signal (Moore, 2012; Moore & Glasberg, 1983; Wong et al., 1998; Zilany et al., 2009; Zwicker, 1970). This would mean that when close to an aircraft taking off, the birds would not be able to perceive any other acoustic signal, irrespective of its spectral structure or amplitude. In conclusion, the decrease in the maximum frequency of chiffchaffs’ songs near the airport is unlikely to be an adaptation to the local soundscape because it does not release the song from masking by aircraft noise.

If it is not an adaptation to the local soundscape, then what may drive the spectral and temporal changes in the songs of chiffchaffs near airports? Several other field studies reported a decrease in spectral or temporal parameters in bird song. Red‐winged blackbirds (*Agelaius phoeniceus*) reduced the delivery rate (Ríos‐Chelén, Lee, & Patricelli, 2015), while great tits (*Parus major*) and white‐crowned sparrows (*Zonotrichia leucophrys nuttalli*) reduced the maximum frequency in their songs in response to noisy conditions (Gentry, Derryberry, Danner, Danner, & Luther, 2017; Halfwerk & Slabbekoorn, 2009). The reduction in spectral characteristics was explained as a strategy to increase the signal‐to‐noise ratio, either through masking release or through the concentration of energy in a narrower frequency bandwidth. However, these strategies would not be effective at the airport. First, the energy in aircraft noise is biased towards the low frequencies, and thus, a reduction in song frequency will not lead to effective release from masking. Second, the soundscape at the airport between aircraft movements, when the recordings were made, is similar to the soundscape of the control population. It is therefore difficult to explain what selection pressure would drive the airport birds to use a narrower frequency bandwidth, but not the birds in the control site.

A decrease in spectral parameters of songs has been observed in several laboratory studies that involve birds with acquired hearing loss. The downward shift in song frequency (±200 Hz) observed in the current study is consistent with the effect observed in zebra finches (*Taeniopygia guttata*) with acquired hearing loss due to long‐term noise exposure (Potvin & MacDougall‐Shackleton, 2015). Similarly, surgically deafened budgerigars (*Melopsittacus undulatus)* and zebra and Bengalese finches (*Lonchura striata)* also sing songs containing more low‐frequency syllables and at a slower rate (Watanabe, Eda‐Fujiwara, & Kimura, 2007; Watanabe & Sakaguchi, 2010) compared to before deafening. In addition, the minimum frequencies in the songs of chiffchaffs exposed to aircraft noise did not change, which is consistent with the song behaviour of birds with laboratory‐induced hearing impairment.

Birds regularly exposed to noise levels of more than 93 dB(A) may suffer from auditory threshold shifts (Dooling & Popper, 2007; Ryals et al., 1999). Although noise events differ in intensity between territories, depending on distance to the runway, flightpath and topographical features (see Figure 2), airport chiffchaffs are exposed to a noise event on average every 180s throughout the day. Chiffchaffs that were exposed to the highest sound levels showed the lowest maximum frequency in their songs, while the control population showed the more commonly observed positive relationship between sound level and song frequency. One potential explanation for the findings of decreased frequency and temporal parameters in the songs is that the chiffchaffs suffer from noise‐induced hearing loss (NIHL).

The downward shift of the maximum frequency in the songs of chiffchaffs at the airport is a result of the songs containing fewer high‐frequency syllables. This suggests that the high‐frequency notes disappear from their repertoire through selective attrition, a process observed in surgically deafened birds (Watanabe & Sakaguchi, 2010). Loud noise exposure, irrespective of frequency content (Yost, 2013), has the greatest impact on high‐frequency hearing (Marler, Konishi, Lutjen, & Waser, 1973), because hair cells located basally in the cochlea, where detection of high‐frequency sounds occurs, are most susceptible to damage (Wang & Ren, 2012). Birds require auditory feedback of their own song to maintain the song's acoustic structure (Nordeen & Nordeen, 1992; Price, 1979; Woolley & Rubel, 1997, 2002) and when unable to detect the higher frequencies in their own song, they stop producing them (Watanabe & Sakaguchi, 2010; Wright, Brittan‐Powell, Dooling, & Mundinger, 2004). Therefore, if chiffchaffs at the airport suffer from reduced sensitivity to high frequencies, they may not be able to hear the higher frequency syllables in their own songs and as a result no longer produce them.

Chiffchaffs exposed to aircraft noise responded more aggressively to simulated territorial intrusions than control birds, similar to other species exposed to anthropogenic noise (Phillips & Derryberry, 2018). In general, urban birds tend to be more aggressive, but whether this is due to noise or other urban factors is not clear (Davies & Sewall, 2016). The current study contributes to the notion that noise may be a prominent factor, since many other aspects of an urban area are not present at an airport. Indeed, the higher levels of aggression may be the direct result of higher stress levels as a result of intermittent aircraft noise exposure, as observed for humans (Stansfeld & Matheson, 2003) and birds (Burger, 1981; Goudie & Jones, 2004) near airports. Harlequin ducks showed increased aggression for 2 hr after a low flying military aircraft passed by (Goudie & Jones, 2004), while in the current study chiffchaffs were exposed to aircraft movement on average every 180s.

The overall high agitation level of airport chiffchaffs may also occur because they are less able to use acoustic information to assess the quality of intruders, due to intermittent noisy conditions. Airport birds were particularly aggressive in response to the airport‐type stimulus, which they demonstrated by attacking the loudspeaker more when it played airport‐type stimuli than when it played control‐type stimuli (Figure 5). One explanation for the higher response to low‐frequency stimuli is that the airport birds are more sensitive to low frequencies due to frequency‐dependent NIHL. The airport stimuli may be perceived as full song stimuli, while the control stimuli, containing more high‐frequency notes, would only be perceived partially (Linhart et al., 2013). However, all playback trials were conducted in between aircraft movements in periods of relative quiet. Therefore, noise cannot have been a direct factor contributing to the difference in response. Although our results show that airport chiffchaffs are able to detect the stimuli, it is possible that as a result of NIHL, they lack the ability to perceive the fine structure of the songs (Lohr et al., 2003). In many bird species, the fine structure of song conveys information about the quality of the singer and determines the individual response of a challenged individual (Ballentine, Hyman, & Nowicki, 2004; de Kort, Eldermire, Valderrama, Botero, & Vehrencamp, 2009b; Podos, 1997). The airport birds may not be able to assess the relative quality of the simulated intruder on the basis of acoustic information and may have to resort to visual displays and physical attack rather than enter into a vocal duel as a first line of defence of their territory.

In conclusion, we have shown that chiffchaffs lower the maximum frequency of their songs and decrease song rate when exposed to aircraft noise, which is consistent with the effects observed in laboratory hearing‐impaired birds. We have also shown that airport birds are more aggressive than control birds. This mirrors studies on humans showing that intermittent and extreme noise exposure can lead to non‐auditory psychological effects such as increased stress levels and aggressive behaviour (Basner et al., 2014). Humans and wildlife do indeed seem to suffer similar consequences from noise exposure (Shannon et al., 2016). These results are timely and add fuel to the debate on the ecological costs of airport expansion around the world.

## AUTHORS’ CONTRIBUTIONS

The study was designed by A.D.W., H.S. and S.R.K. Fieldwork was conducted by A.D.W. and S.R.K., with A.D.W. and S.R.K. processing the data. All authors contributed to writing the paper and all authors gave final approval for publication.

## Data Availability

Data available from the Dryad Digital Repository: http://dx.doi.org/10.5061/dryad.3nd978g (Wolfenden, Slabbekoorn, Kluk, & Kort, 2019).
