# Supplementary material for: Aircraft sound exposure leads to song frequency decline and elevated aggression in wild chiffchaffs
Source: J Anim Ecol. 2019 Aug 21;88(11):1720–31. doi: 10.1111/1365-2656.13059 (PMC8647924; doi:10.1111/1365-2656.13059)
Supplement: Supplementary file 1 [file JANE-88-1720-s001.docx]

Online supplementary material.

| Territory i.d. | Latitude | Longitude | Distance to runway (m) | LAFmax dB(A) | LAeq (10) dB(A) | Lden  dB(A) |
| --- | --- | --- | --- | --- | --- | --- |
| CHIFF-144143 | 53.3298 | -2.3084 | 186 | 118 | 61 | 75 |
| CHIFF-263145 | 53.3318 | -2.3055 | 338 | 101 | 56 | 70 |
| CHIFF-244142 | 53.3342 | -2.2991 | 344 | 115 | 64 | 70 |
| CHIFF-173144 | 53.3407 | -2.2876 | 405 | 101 | 63.5 | 70 |
| CHIFF-193143 | 53.3374 | -2.3053 | 518 | 98 | 62 | 70 |
| CHIFF-183144 | 53.3407 | -2.3003 | 520 | 104 | 65 | 70 |
| CHIFF-263146 | 53.3278 | -2.3207 | 554 | 87 | 60 | 70 |
| CHIFF-263147 | 53.3351 | -2.3108 | 640 | 94.1 | 63 | 70 |
| CHIFF-174145 | 53.3407 | -2.3003 | 674 | 88 | 61 | 70 |
| CHIFF-35147 | 53.3276 | -2.3105 | 704 | 83 | 67 | 70 |
| CHIFF-174144 | 53.3294 | -2.3102 | 724 | 76 | 56 | 70 |
| CHIFF-174146 | 53.3446 | -2.2957 | 725 | 76 | 56 | 70 |
| CHIFF-183143 | 53.3403 | -2.3011 | 728 | 82 | 65.4 | 70 |
| CHIFF-173143 | 53.3391 | -2.3031 | 802 | 96 | 55 | 70 |
| CHIFF-174141 | 53.3352 | -2.3247 | 840 | 79 | 58 | 70 |
| CHIFF-313148 | 53.3656 | -2.6313 | 855 | 80 | 54 | 70 |
| CHIFF-313147 | 53.3666 | -2.6260 | 955 | 83 | 60 | 70 |
| CHIFF-174147 | 53.3675 | -2.6213 | 956 | 79 | 58 | 70 |
| CHIFF-193142 | 53.3683 | -2.6157 | 965 | 99 | 64 | 70 |
| CHIFF-173142 | 53.3697 | -2.6136 | 979 | 70 | 61.9 | 70 |
| CHIFF-244141 | 53.3705 | -2.6187 | 1092 | 83.5 | 59 | 70 |
| CHIFF-35148 | 53.3652 | -2.6418 | 1122 | 73 | 54 | 70 |
| CHIFF-173141 | 53.3620 | -2.6483 | 1152 | 83 | 54.2 | 70 |
| CHIFF-144147 | 53.3429 | -2.2921 | 1160 | 74 | 54 | 70 |
| CHIFF-263142 | 53.3546 | -2.3091 | 1212 | 83 | 61.9 | 70 |
| CHIFF-263141 | 53.3527 | -2.3003 | 1401 | 81 | 57.6 | 70 |
| CHIFF-35145 | 53.3363 | -2.3299 | 1502 | 72 | 53 | 70 |
| CHIFF-174148 | 53.3373 | -2.3320 | 1519 | 67 | 55 | 70 |
| CHIFF-144146 | 53.3385 | -2.3346 | 1698 | 77 | 52 | 70 |
| CHIFF-313141 | 53.3392 | -2.3358 | 1710 | 82 | 53.9 | 70 |
| CHIFF-193141 | 53.3420 | -2.3087 | 1761 | 101.7 | 63 | 70 |
| CHIFF-35141 | 53.3405 | -2.3297 | 1826 | 77 | 56 | 70 |
| CHIFF-284141 | 53.3673 | -2.6399 | 1880 | 76.1 | 66 | 70 |
| CHIFF-313142 | 53.3676 | -2.6428 | 2023 | 75 | 51 | 70 |
| CHIFF-313144 | 53.3640 | -2.6580 | 2152 | 70 | 53 | 70 |
| CHIFF-313145 | 53.3609 | -2.6588 | 2324 | 79 | 57 | 70 |
| CHIFF-313146 | 53.3589 | -2.6613 | 2407 | 86 | 59 | 70 |
| CHIFF-35142 | 53.3578 | -2.6619 | 2424 | 74 | 52 | 70 |
| CHIFF-55145 | 53.3581 | -2.6594 | 16248 | 62 | 44.1 | 70 |
| CHIFF-55144 | 53.3601 | -2.6516 | 16380 | 54 | 45 | 70 |
| CHIFF-234146 | 53.3620 | -2.6492 | 16513 | 49.2 | 43.8 | 70 |
| CHIFF-55143 | 53.3395 | -2.2798 | 16555 | 55.6 | 48 | 70 |
| CHIFF-254149 | 53.3367 | -2.2897 | 16616 | 54.5 | 49.5 | 70 |
| CHIFF-55146 | 53.3373 | -2.2893 | 16648 | 50 | 45.4 | 70 |
| CHIFF-254148 | 53.3360 | -2.2926 | 16661 | 55.1 | 51 | 70 |
| CHIFF-254147 | 53.3337 | -2.2923 | 17140 | 59.8 | 42.9 | 70 |
| CHIFF-254146 | 53.3377 | -2.2673 | 17462 | 58.1 | 43.8 | 70 |
| CHIFF-254145 | 53.3648 | -2.6417 | 17521 | 57.9 | 46.6 | 70 |
| CHIFF-254144 | 53.3838 | -2.5376 | 17597 | 61.8 | 47.1 | 70 |
| CHIFF-254143 | 53.3343 | -2.3137 | 17712 | 58.6 | 46.1 | 70 |
| CHIFF-104147 | 53.3440 | -2.2960 | 21070 | 50 | NA | 65 |
| CHIFF-104146 | 53.3296 | -2.3249 | 21183 | 63 | NA | 65 |
| CHIFF-104149 | 53.3548 | -2.3116 | 21417 | 53 | NA | 65 |
| CHIFF-104145 | 53.3595 | -2.3164 | 21539 | 53 | NA | 65 |
| CHIFF-104144 | 53.3525 | -2.3084 | 21835 | 65 | NA | 65 |
| CHIFF-104142 | 53.3413 | -2.3041 | 22161 | 56 | NA | 65 |
| CHIFF-154141 | 53.3509 | -2.3029 | 22755 | 57 | NA | 65 |
| CHIFF-234142 | 53.3883 | -2.5358 | 22837 | 55.9 | NA | 65 |
| CHIFF-114142 | 53.3869 | -2.5337 | 22850 | 67.3 | NA | 65 |
| CHIFF-154142 | 53.3863 | -2.5320 | 22952 | 58 | NA | 65 |
| CHIFF-114143 | 53.3849 | -2.5392 | 23242 | 54 | NA | 65 |
| CHIFF-154149 | 53.3948 | -2.5503 | 23297 | 57 | NA | 65 |
| CHIFF-154148 | 53.3947 | -2.5485 | 23432 | 55 | NA | 65 |
| CHIFF-154143 | 53.3938 | -2.5479 | 23906 | 62 | NA | 65 |
| CHIFF-154146 | 53.3930 | -2.5474 | 23906 | 58 | NA | 65 |
| CHIFF-154144 | 53.3922 | -2.5428 | 23918 | 51 | NA | 65 |
| CHIFF-154147 | 53.3933 | -2.5343 | 23930 | 58 | NA | 65 |
| CHIFF-154145 | 53.3926 | -2.5341 | 24067 | 64 | NA | 65 |

Table 1. Coordinates of centre point of chiffchaff territories at Manchester airport and the control population. Those at a distance of less than 2.5km are airport territories, those over 16km are control territories. Sound level values for each territory are: LAFmax = the maximum level A-weighted fast response; LAeq (10) = the average level over 10 minutes, A-weighted, fast response; Lden = sound level band reported by the MANTIS system at Manchester airport. Lden is a descriptor of noise level based on energy equivalent noise level (Leq) over a whole day with a penalty of 10 dB(A) for night time noise (22.00-7.00) and an additional penalty of 5 dB(A) for evening noise (i.e. 19.00-23.00)
